# Intraoperative Coronary Spasm: A Potential Case of Vasospastic Angina

**DOI:** 10.7759/cureus.44561

**Published:** 2023-09-02

**Authors:** Pedro Camões Correia, Ana Leite, Pedro Alves Marques, Teresa Lugarinho

**Affiliations:** 1 Anesthesiology, Centro Hospitalar Universitário de Coimbra, Coimbra, PRT

**Keywords:** carotid endarterectomy, myocardial ischemia and infarction, cardiac arrest outcome, vascular hyperreactivity, prinzmetal’s angina

## Abstract

Prinzmetal’s angina typically features spasms of the coronary arteries due to the hyperreactivity of the vascular smooth muscle cells of the vessels to a nonspecific stimulus. Reports of coronary spasm during general anesthesia are rare, but in such cases, diagnosis is suggested by a framework of angina at rest and changes in the electrocardiogram (ECG) or coronary reactivity tests with ergonovine or acetylcholine. The present study describes a case of coronary spasm induced by general anesthesia associated with several cardiovascular risk factors and the usage of vasoactive drugs that was documented by angiography without using stimulating drugs and treated with intracoronary nitroglycerin.

The patient was a 58-year-old male who was designated for carotid endarterectomy due to the stenosis (70%) of the right internal carotid artery by an atheromatous plaque after visiting the emergency department with a sensorimotor deficit in the left upper limb and bifrontal headaches with sudden onset. During the surgical intervention, after the administration of 10 mg of intravenous ephedrine, cardiorespiratory arrest occurred, with alternation between defibrillable and non-defibrillable heart paces. After the recovery of spontaneous circulation after 50 minutes of resuscitation maneuvers, the patient was transported to the hemodynamics laboratory, where there were recurrent episodes of ventricular fibrillation during the angioplasty of the anterior descending artery. After direct stent implantation, pre- and post-stent spasms were verified and reversed after the administration of intracardiac nitroglycerin.

The spasm was a possible complication of anesthesia and responded to treatment with nitrates and calcium channel blockers. We would like to emphasize the importance of cardiac monitoring during surgery and anesthesia.

## Introduction

Prinzmetal’s angina, also known as variant angina or vasospastic angina, is a clinical condition first described by Prinzmetal that is characterized as chest discomfort or pain at rest with transient electrocardiographic changes in the ST segment and with a prompt response to nitrates by spasms of the coronary arteries. It may occur in angiographically normal coronary arteries or with associated atherosclerotic pathology, and it is caused by a localized hyperreactivity of the smooth muscle cells of the vessels to a nonspecific stimulus [1-3]. Although reports of coronary spasm during general anesthesia are scarce (<0.1%), in those reported, the diagnosis is suggested by angina at rest and changes in the electrocardiogram (ECG) or coronary reactivity tests with ergonovine or acetylcholine [1,4,5]. Here, we report a case of coronary spasm induced by general anesthesia that was diagnosed by angiography without the use of stimulation drugs and treated with intracoronary nitroglycerin.

## Case presentation

We report a case of a 58-year-old Caucasian male patient who was admitted to the emergency department of our hospital due to sensory-motor deficit in the left upper limb and bifrontal headaches with sudden onset. The patient had uncontrolled cardiovascular risk factors, namely, type 2 diabetes mellitus, hypertension, and dyslipidemia (all diagnosed during hospitalization), and he was also a smoker. He weighed 92 kg and was 163 cm tall. His body mass index was 35 kg/m^2^. The patient was taking atorvastatin 20 mg and ramipril 1.25 mg.

In the emergency department, the patient underwent a computerized scan of the brain, which revealed left posterior and internal lenticular hypodensity, possibly with slight involvement of the posterior arm of the internal capsule, with no associated mass effect, and likely translating subacute ischemic injury; he also underwent a cervical and transcranial echocardiogram-Doppler, which showed a stenosis of 70% of the right internal carotid artery by an atheromatous plaque. Laboratory tests showed creatinine of 0.71 (0.3-1.1), Na of 140 (135-145), K of 4.2 (3.5-5.5), troponin of 0.053 (<0.014), hemoglobin of 13.5 (12-14), platelets of 256,000 (150,000-450,000), total cholesterol (Col T) of 280 (<200) with low-density lipoprotein (LDL) of 180 (<110), ECG sinus rhythm at 76 beats per minute (bpm), and glycated hemoglobin (HbA1C) of 7.2 (<6). The echocardiogram showed a normal left ventricular ejection fraction of 52% with no regional wall motion abnormalities. The patient underwent an elective right carotid endarterectomy in view of his symptoms and the severity of the lesion five days later.

On the day of the procedure, his blood pressure (BP) and heart rate (HR) were so 145/62 mmHg and 82 beats/minute, respectively. ECG showed a normal sinus rhythm without signs of atrioventricular blocks and T wave and ST segment without alterations, and oxygen saturation (SpO_2_) was 98%.

A balanced general anesthesia was performed. The patient was induced with 50 μg of fentanyl, 500 mg of thiopental, and 35 mg of atracurium with adequate anesthetic depth; subsequently, this was followed by an orotracheal intubation. Anesthesia was maintained with 1%-2% sevoflurane and intermittent fentanyl injection. Anesthetic depth was controlled via the bispectral index (BIS). The standard monitoring parameters of the American Society of Anesthesiologists were used, complemented by invasive blood pressure in the left radial artery.

After intubation and adaptation to a ventilator, the patient had the following vitals: saturation of 99% with the fraction of inspired oxygen (FiO_2_) at 40%, BP of 110/56, body temperature of 36.5°C, and glycemia of 120.

During surgical intervention, after 10 minutes of intubation to correct a slight hypotension (90 systolic) and after anesthetic induction, 10 mg of intravenous ephedrine was administered when the cardiorespiratory arrest occurred, detected through the absence of pulse at the carotidal level and the absence of a pulse curve on the monitor for invasive arterial monitorization (with pulseless electrical activity). An immediate request for assistance ensued, and advanced life support maneuvers were immediately initiated (following advanced cardiac life support {ACLS} protocols): 1 mg of intravenous adrenaline was administered, and oxygen was supplied with an inspiratory fraction of 100%. The patient’s heart pace varied between defibrillable (ventricular fibrillation) and non-defibrillable, and the resuscitation maneuvers lasted for 50 minutes, with the eventual recovery of the spontaneous circulation of the patient in sinus rhythm.

Following the discussion of the case with the cardiology team, it was decided to send the patient to the hemodynamics laboratory under noradrenaline infusion. The anesthetic team escorted him, with ventilator support. 

**Figure 1:**
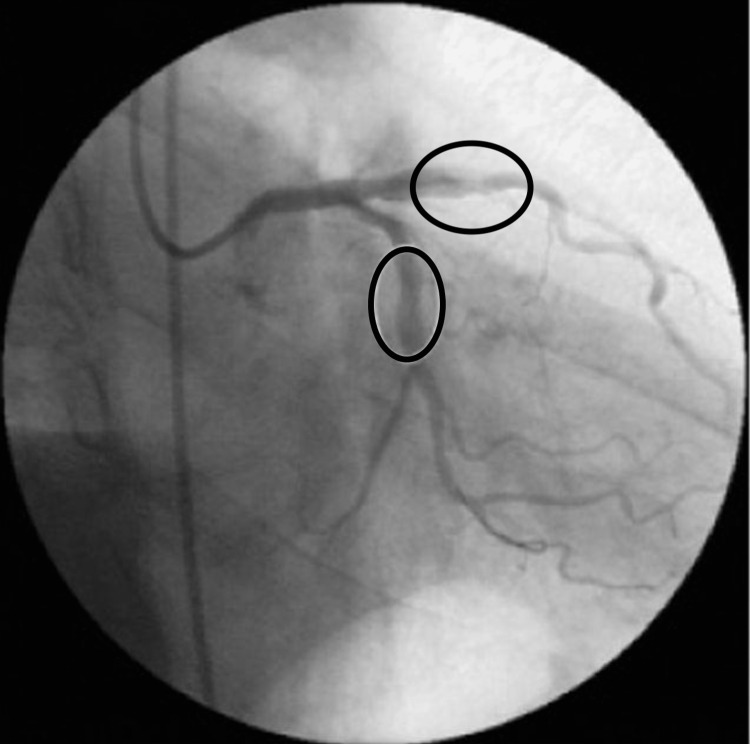
Coronary angiography with a 75% lesion in the anterior descendant and a lesion of less than 50% in the middle portion of the circumflex artery.

**Figure 2:**
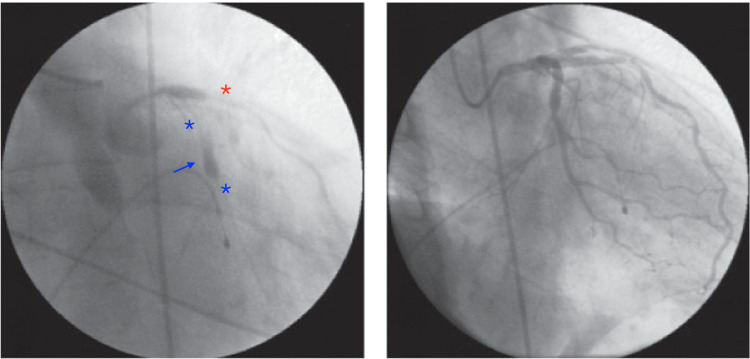
The blue arrow shows the placement of the stent, with spasm before and after the stent (blue stars) and slightly (red star) in the circumflex artery (right image), which reversed after the administration of intracardiac nitroglycerin (left image).

A coronary angiography was performed, revealing a short common trunk of the left coronary artery, without injuries, unlike the anterior descending artery that showed a 75% occlusion involving its origin. The first diagonal artery (D1) was of very small caliber and presented a total occlusion in its middle portion. Finally, the circumflex artery presented a <50% occlusion in its middle portion. During the angioplasty of the proximal anterior descending artery, there were recurrent episodes of ventricular fibrillation, which reversed after shocking with 250 J. Coronary angioplasty was performed, with the placement of a proximal pharmacological stent. After the implantation, there were artery spasms before and after the stent. Slight spasms were also identified in the circumflex artery, but these reversed after the administration of intracoronary nitroglycerin (Figures 1, 2).

Subsequently, the patient was transferred to the intensive coronary care unit (ICCU), where he remained sedated, intubated, and ventilated until the second day of hospitalization. Extubation was done on the fourth day of hospitalization at the ICCU.

As a follow-up for the cardiocirculatory arrest, a transthoracic echocardiogram was performed, which revealed good global systolic function. Meanwhile, computed axial tomography and cranioencephalic magnetic resonance imaging revealed injury to the posterior arm of the left internal capsule. An electroencephalogram showed the diffusely slowing of the background with predominant theta/delta waveform.

The patient was later transferred to a neurological rehabilitation unit. At the time of discharge, the patient was vigil and stable, with mixed transcortical aphasia and the spontaneous mobilization of the four limbs with bilateral ideomotor apraxia, without focal motor deficits. Since then and up to the time of writing this article, the patient has been followed in cardiology and neurology to keep tabs on risk factors, therapeutic adjustments, and clinical progress.

## Discussion

In this clinical case, there was a coronary vasospasm in a patient with undiagnosed Prinzmetal’s angina, which was not brought upon by excessive activity of the heart but happened spontaneously at rest or in light and common daily activities.

Prinzmetal’s angina happens with a significant elevation of the T segment and the reciprocal depression of the ST segment on the ECG [1,5].

Nevertheless, the recognition of variant angina is not always easy during preoperative evaluation, and it may even prove impossible in asymptomatic patients with a normal ECG [2]. Usually, this clinical condition occurs in younger individuals with lesser cardiovascular risk and may be confirmed by medically provoking a local vasospasm by vasoconstrictors such as ergonovine and acetylcholine during percutaneous transluminal coronary angioplasty (PTCA) [1,3,4].

The condition is more prevalent in the Japanese population (three times as much as the Caucasian population) [6,7], and it has been implicated as a cause of syncope, tachyarrhythmias, acute myocardial infarction, and sudden death [1]. Prinzmetal’s angina is associated with spontaneous coronary dissection by the stimulation of alpha and beta vascular receptors, with vasoconstriction and associated arterial hypertension, which increases the shear stress in the arterial wall and may result in arterial dissection [3].

Although the cause of coronary spasm remains unknown, altered humoral factors, the increase in catecholamines associated with anesthesia and surgery, excess alpha-adrenergic activity, the stimulation of the parasympathetic nervous system, and imbalance between the vasoconstriction-vasodilation mediators are considered possible mechanisms [4]. Although the coronary arteries involved are often normal, it is possible that they are affected by atherosclerosis, with endothelial dysfunction.

Sevoflurane and desflurane have primary vasodilatory properties, thereby reducing systemic vascular resistance (SVR). Desflurane tends to produce progressive tachycardia at progressively higher concentrations, and tachycardia may be seen with sevoflurane at concentrations >1.0 minimum alveolar concentration (MAC).

In particular, desflurane may induce tachycardia and hypertension due to its sympathomimetic properties, particularly with high or abruptly increased concentrations.

In this case, we used sevoflurane, which presented no theoretical risk for sensitivity to catecholamines, like other volatile anesthetics [8-13]. The cardiorespiratory arrest was in a non-defibrillable rhythm, with subsequent alternation between defibrillable (ventricular fibrillation) and non-defibrillable rhythms. The adrenaline administered intravenously during the cardiorespiratory resuscitation maneuvers may have aggravated the coronary vasospasm [2,9].

During general anesthetics, the clinical information of the patient is lost, making it impossible to evaluate the characteristic symptoms of Prinzmetal’s vasospastic angina. Therefore, at intrasurgical level, the only way to early diagnose this clinical condition is to have an ECG with an elevation in the S segment with reciprocal depression in the ST segment. Thus, the continuous vigilance of the patient at the intraoperative level with standard monitorization is absolutely necessary for the early detection of Prinzmetal’s vasospastic angina. It is important to state that Prinzmetal’s angina may not be noticeable even with coronary vasospasm during surgery.

There are several drugs used for anesthetics that may promote coronary vasospasm, such as ephedrine, phenylephrine, noradrenaline, and adrenaline [9].

In the present study, the patient presents hypotension post induction before commencing surgical procedures, which probably did not originate from the coronary spasm. To reverse the hypotension, 10 mg ephedrine was used to hemodynamically stabilize the patient before the start of the surgical procedure, which may have triggered the coronary vasospasm. It is possible that this alpha receptors’ agonist may have provoked coronary spasm through the activation of the autonomic nervous system. In addition, an atheromatous plaque in the anterior descending coronary artery, later diagnosed on angiography, may have made this artery more susceptible to spasm.

We need to remember that the coronary vasospasm may have had other potential causes, such as beginning the surgical procedures without adequate anesthetic depth, the usage of certain drugs, hemodynamic instability, or even cardiovascular risk factors presented by the patient [8,9,14,15].

The treatment of Prinzmetal’s angina consists of administering fluid therapy and inotropes such as dobutamine for good hemodynamic control. In this patient, coronary spasm was detected by coronary angiography without the need for ergonovine or acetylcholine (i.e., drugs used in provocative tests during coronary angiography). According to the literature, the definitive diagnosis was made using both coronary angiography and treatment with intracoronary nitroglycerin [16-20]. The recommended treatment for variant angina consists of nitrates and calcium channel blocker (diltiazem 240-360 mg or amlodipine 5-10 mg daily) [4,6,15,16,19,20], which are extremely effective in the prevention and treatment of coronary vasospasm. In the present case, we used intracoronary nitroglycerin, with the resolution of the coronary spasm. Nitrates have their pharmacological effect regardless of vascular endothelial cells, and calcium channel blockers work directly on vascular smooth muscle [8,15]. Likewise, current literature suggests that these drugs have better results in reverting coronary spasm when there is no atherosclerosis [9]. Thus, the early administration of these drugs is essential.

Given the cardiovascular risk factors presented by the patient, the initial most probable clinical hypothesis was atherosclerotic coronary disease. In the present study, we demonstrated that in pre-surgery, the patient did not show any clinical issues regarding chest pain and had done complementary diagnostic tests such as ECG, blood tests, and echocardiogram, which had no significant cardiac chances, just dyslipidemia and diabetes, both properly medicated during admission.

Despite the risk factors present for cardiovascular disease, namely, hypertension, dyslipidemia, and carotidal atherosclerosis, and as the preoperative thoracic echocardiogram was normal, the possibility of an underlying cardiac disease was considered moderate. Therefore, Prinzmetal’s angina was not suspected, and ephedrine was used with no notion of its potential risk for coronary spasm.

In fact, the literature suggests that Prinzmetal’s angina is equally frequent in both males and females but more frequent in ages under 50, in the Japanese population, and in patients with fewer cardiovascular risks [6]. Taking all this into account, we believe that it is important to present this case study because it does not include any of the aforementioned variables.

Not of less importance, a post-surgery assessment or the reversing of the acute episode should be carried out, to achieve an accurate diagnosis and so that the patient may receive necessary treatment.

Patients that present this clinical condition should be given information so as to explain their clinical situation to future anesthesiologists when facing eventual surgery. Thus, the anesthetist can plan the procedure with strategies that will avoid an episode of vasospasm (not using certain drugs, for instance), and if such episode should occur, they can allow for a faster diagnosis and a more effective and directed treatment.

## Conclusions

As has been previously shown, Prinzmetal’s angina may result in fatal arrhythmia and myocardial ischemia, even with no early changes in ECGs. At the intrasurgical stage, the anesthetist must be aware of the risks of the occurrence of this clinical condition, adapting their anesthetic procedures. Likewise, Prinzmetal’s angina may not be easily detected during the anesthetic process because the symptoms and typical signs are hidden. This means that diligent vigilance by anesthesiologists and an immediate response and adequate treatment are essential.

This article described a case of coronary spasm during general anesthesia documented immediately using angiography without the use of pharmacological stimulation and treated with intracoronary nitrates. The spasm was a possible complication of the anesthesia and cardiovascular risks and responded to treatment with nitrates and calcium channel blockers. We would also like to emphasize the importance of cardiac monitoring during surgery and anesthesia use. It is important to note that the aforementioned clinical case is thought to be of extreme relevance for the current scientific literature, as it shows a case that does not include the usual patient profile associated with vasospastic Prinzmetal’s angina.
